# Post-psychotic depression: what are its characteristics?

**DOI:** 10.1192/j.eurpsy.2024.1098

**Published:** 2024-08-27

**Authors:** A. Abdelhamid, S. Bellafqih, F. Laboudi, A. Ouanass

**Affiliations:** Psychiatry, Ar-Razi Psychatric Hospital, Sale, Morocco

## Abstract

**Introduction:**

Depression in psychosis has been more or less neglected as a field of study, due to its vague nosographic framework. Some studies have nevertheless focused on certain features of depression in psychosis, such as post-psychotic depression. This is a frequent phenomenon with a nosographic and etiopathogenic complexity that can lead to confusion.

**Objectives:**

To study the characteristics of post-psychotic depression and compare results with those in the literature.

**Methods:**

It is a prospective, descriptive, case series study conducted at the Ar-Razi psychiatric hospital in Salé. Inclusion criteria were patients diagnosed with a brief psychotic disorder, schizophreniform disorder or schizophrenia, in remission, who met the criteria for a DSM 5 characterized depressive episode. Data are collected during the psychiatric interview with the patient, using a questionnaire.

**Results:**

Ongoing

**Conclusions:**

Ongoing

**Disclosure of Interest:**

None Declared

